# High Fructose Intake During Pregnancy in Rats Influences the Maternal Microbiome and Gut Development in the Offspring

**DOI:** 10.3389/fgene.2018.00203

**Published:** 2018-06-19

**Authors:** Stuart Astbury, Aleida Song, Mi Zhou, Brent Nielsen, Abha Hoedl, Benjamin P. Willing, Michael E. Symonds, Rhonda C. Bell

**Affiliations:** ^1^Division of Human Nutrition, Department of Agricultural, Food and Nutritional Science, University of Alberta, Edmonton, AB, Canada; ^2^Early Life Research Unit, Division of Child Health, Obstetrics and Gynaecology, School of Medicine, The University of Nottingham, Nottingham, United Kingdom; ^3^Nottingham Digestive Diseases Centre, School of Medicine, University of Nottingham, Nottingham, United Kingdom; ^4^NIHR Nottingham Biomedical Research Centre, University of Nottingham and Nottingham University Hospitals NHS Trust, Nottingham, United Kingdom; ^5^Division of Animal Science, Department of Agricultural, Food and Nutritional Sciences, University of Alberta, Edmonton, AB, Canada; ^6^Women and Children’s Health Research Institute, University of Alberta, Edmonton, AB, Canada

**Keywords:** fructose, pregnancy, nutrition, microbiome, diabetes

## Abstract

Studies in pregnant women indicate the maternal microbiome changes during pregnancy so as to benefit the mother and fetus. In contrast, disruption of the maternal microbiota around birth can compromise normal bacterial colonisation of the infant’s gastrointestinal tract. This may then inhibit development of the gut so as to increase susceptibility to inflammation and reduce barrier function. The impact of modulating fructose intake on the maternal microbiome through pregnancy is unknown, therefore we examined the effect of fructose supplementation on the maternal microbiome together with the immediate and next generation effects in the offspring. Wistar rat dams were divided into control and fructose fed groups that received 10% fructose in their drinking water from 8 weeks of age and throughout pregnancy (10–13 weeks). Maternal fecal and blood samples were collected pre-mating (9 weeks) and during early (gestational day 4–7) and late pregnancy (gestational day 19–21). We show supplementation of the maternal diet with fructose appears to significantly modulate the maternal microbiome, with a significant reduction in *Lactobacillus* and *Bacteroides*. In offspring maintained on this diet up to pregnancy and term there was a reduction in gene expression of markers of gut barrier function that could adversely affect its function. An exacerbated insulin response to pregnancy, reduced birth weight, but increased fat mass was also observed in these offspring. In conclusion dietary supplementation with fructose modulates the maternal microbiome in ways that could adversely affect fetal growth and later gut development.

## Introduction

The intestinal microbiota is an important mediator of human metabolic health that can determine the onset of obesity and the metabolic syndrome (Turnbaugh et al., 2006). Dietary composition, rather than host genetics, can have a dominant role in modulating the microbiome (Carmody et al., 2015). This relationship could be especially important during pregnancy when the maternal microbiome can undergo pronounced changes which are not dissimilar to those seen with obesity, becoming more pro-inflammatory and capable of inducing metabolic inflammation (Koren et al., 2012). Disruption of the maternal microbiota can affect microbial colonization in the offspring, potentially leading to increased intestinal permeability and reduced stomach growth (Fåk et al., 2008).

Although the impact of maternal diet (Payne et al., 2012; Cotillard et al., 2013; David et al., 2014), and specific dietary constituents (Gohir et al., 2015) on the microbiome has been examined, little is known about the impact of specific carbohydrates such as fructose. Globally, increased fructose intake has paralleled the prevalence of obesity and is widely recognized as a primary carbohydrate contributing to the rise in caloric consumption in Western diets (Lim et al., 2010). Although fructose in the form of high fructose corn syrup has been the focus of media and research attention (Bray et al., 2004; Goran et al., 2013), excessive consumption of fruit juices that are rich in fructose is also a public health concern (Faith et al., 2006). Fructose is metabolized differently to glucose, with the majority diverted toward hepatic lipogenesis, and has no effect on insulin release (Bezerra et al., 2000) while suppressing ghrelin secretion (Havel, 2005). Consumption of a high-fructose diet can induce insulin resistance (Bezerra et al., 2000; Huang et al., 2004), hypertension (Hwang et al., 1987), and dyslipidemia (Kazumi et al., 1997) in adult rodents.

Excess caloric intake in the form of a high-fat diet can adversely affect intestinal permeability, allowing components of the microbiota to pass into the systemic circulation and contribute to inflammation of adipose tissue (Lam et al., 2011). This type of chronic inflammation has subsequently been linked to the development of the metabolic syndrome (Lam et al., 2011). The impact of fructose on this process in the mother or offspring is unknown.

We have recently shown that although feeding a high fructose diet modulates the mother’s metabolism, it has little impact on the offspring (Lineker et al., 2016) despite being smaller at birth. However, the maternal response appears to be amplified in the next generation when maintained on the same fructose supplemented diet (Song et al., 2017). Other groups have demonstrated that a high fructose intake during pregnancy will affect fetal endocrine function (Vickers et al., 2011) and lipid metabolism (Clayton et al., 2015). Young pigs and rats showing intrauterine growth restriction have a small intestine that is disproportionately affected with a reduced surface area (Younoszai and Ranshaw, 1973; Lebenthal et al., 1981; Xu et al., 1994) but effects on the microbiome remain to be defined. The aim of the present study was to determine whether pregnancy impacted on the maternal microbiome and was modifiable by fructose feeding. In addition, we examined whether female offspring born to dams fed a fructose-supplemented diet would show persistent differences within their small intestine that could further compromise glucose homeostasis in the next generation.

## Materials and Methods

### Animal Study

The study protocol was approved by the Research Ethics Office of the University of Alberta, in accordance with regulations set by the Canadian Council on Animal Care.

Two generations of Wistar rats were used. Rats were obtained from Charles River Laboratories (Montreal, QC, Canada) at 7 weeks of age, and were allowed *ad libitum* access to chow (LabDiet 5001, Purina, MI, United States) and distilled water. At 8 weeks, female rats were randomly assigned to receive either 10% fructose solution or continue receiving distilled water. Rats were mated at 10 weeks of age and diets continued through pregnancy.

**FIGURE 1:**
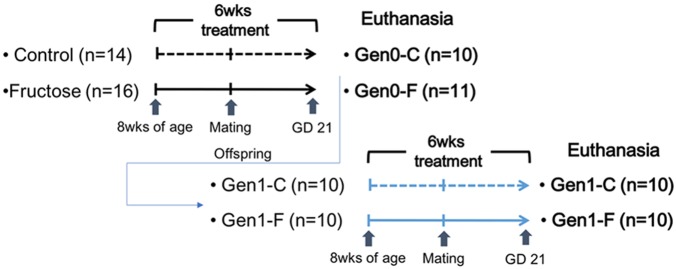
Outline of the animal study.

At gestational day (GD) 20, 10 control (Gen0-C) and 11 fructose-fed (Gen0-F) dams were euthanized using CO_2_ for tissue collection. The remaining dams in each group were left to litter out and continued to receive their respective diets during lactation. Pups were placed on the control diet at weaning, until being placed on the same diet as their dam at 8 weeks of age, receiving either the 10% fructose (Gen1-F, *n* = 10) or distilled water (Gen1-C, *n* = 10). These diets continued through mating at 11 weeks and throughout pregnancy. Second generation dams were euthanized at GD20 for tissue collection. All rats from both generations were imaged via MRI (Echo Medical Systems, Houston, TX, United States) immediately prior to euthanasia for fat and lean mass measurements. An outline of the animal study is given in **Figure 1**.

### Sample Collection and Measurement

All rats were weighed weekly. Blood samples were taken at four timepoints during the study: pre-diet (PD, 7 weeks), pre-mating (PM, 9 weeks), early pregnancy (EP, GD 4–7), and mid pregnancy (MP, GD 14–17). Whole blood was obtained from the tail vein and collected in K_2_ EDTA microtainer tubes (BD, Franklin Lakes, NJ, United States). Plasma was separated from whole blood via centrifugation immediately following collection and stored at -20°C until analysis. Plasma glucose was determined via glucose (Trinder) assay (Genzyme Diagnostics, Charlottetown, PE, Canada). Plasma insulin concentrations were measured using the Insulin (Rat) Ultrasensitive ELISA Immunoassay kit (ALPCO Diagnostics, Salem, NH, United States). Plasma triglyceride concentrations were determined using the Triglyceride-SL assay kit (Genzyme Diagnostics).

Oral glucose tolerance tests (OGTTs) were carried out using 3 g glucose per kg of body weight administered by gavage to pregnant rats following a 4-h fast on GD19 [late pregnancy (LP)]. Blood samples were collected from the tail vein at 0, 15, 30, 45, 60, and 90 min intervals following administration of the oral glucose. Plasma glucose and insulin during OGTT were determined using the methods described above. OGTT results are presented as concentrations at each timepoint and incremental area under the curve (IAUC).

At dissection, the length of the small intestine (duodenum to cecum) was measured, and flushed with cold 10% phosphate buffered saline (PBS). Sections of ileum and jejunum were snap-frozen in liquid nitrogen for protein and RNA extraction. All snap-frozen tissue was transferred to a -80°C freezer for storage. Sections of ileum and jejunum were also fixed in buffered zinc formalin (Z-Fix, Anatech Ltd., Battle Creek, MI, United States) for histology.

### RNA Extraction and Quantitative PCR

A total of 60–80 mg of snap-frozen intestine and liver was homogenized in Tri-reagent (Ambion Diagnostics, Austin, TX, United States), and RNA extraction was carried out using the Qiagen RNeasy Mini Kit (Qiagen N.V., Hilden, Germany). RNA concentration and purity was confirmed via Nanodrop (Thermo Scientific, Waltham, MA, United States), and reverse transcription PCR carried out using the High Capacity cDNA reverse transcription kit (Applied Biosystems, Carlsbad, CA, United States). qPCR was then carried out using SYBR Green dye and the StepOne Plus PCR machine (Applied Biosystems). Primers were designed using Primer3 (Untergasser et al., 2007). All primers were designed to be intron-spanning to avoid amplification of genomic DNA. All qCPR results were adjusted to two reference genes (RPLP0 and GAPDH), using GeNorm software (Vandesompele et al., 2002) and are presented as fold change relative to the control group, calculated using the 2-ddCt method (Livak and Schmittgen, 2001). All sequences are included in Supplementary Table S1.

### Histology

Approximately 2.5 cm length sections of ileum and jejunum were fixed in Z-Fix at dissection after flushing with cold 10% PBS. Four transverse sections of each sample were cut and embedded in paraffin blocks before sectioning at 4 μm and mounting, and were stained using hematoxylin and eosin. Villus height measurements were made using ImageJ, taking an average of measurements from 10 fields per section at 40x magnification.

### Statistics

Glucose, triglyceride, insulin, and qPCR data were analyzed as follows. The four groups were compared using one-way analysis of variance (ANOVA) using contrast coefficients to compare both diet and generation or Kruskal–Wallis with *post hoc* tests depending on distribution. Data distribution was assessed with the Shapiro–Wilk test. All statistical analyses were carried out in SPSS v.21 (IBM Corp., Armonk, NY, United States) and presented using Prism 6 (GraphPad Software Inc., La Jolla, CA, United States).

### Fecal DNA Extraction, Pyrosequencing, and Data Analysis

Fecal samples were collected at PM, EP, and LP and stored at -20°C until extraction; 180–220 mg of each sample was homogenized in a bead beater homogenizer (FastPrep 24, MP Biomedicals, Solon, OH, United States) and DNA extraction carried out using the QIAmp DNA Stool Mini Kit (Qiagen), following the standard protocol. The extracted DNA samples were quantified using a Nanodrop spectrophotometer (Thermo Fisher).

Universal 16s rRNA primers (27F and 519R) were used to generate amplicons from each fecal DNA sample. Each 20 μL reaction contained 0.4 U of Phusion Hot Start II High-Fidelity DNA Polymerase (Thermo Scientific), 1 × Phusion HF Buffer, 8 pmol of each primer, 4 mM dNTP, and 25 ng of DNA template. PCR was carried out under the following conditions: initial denaturing at 98°C for 1 min, followed by 30 cycles at 98°C for 10 s, 59°C for 30 s, and 72°C for 30 s, and a final extension at 72°C for 7 min. The PCR products were run on 1% agarose gels and excised using a scalpel. Agarose gel extractions were carried out using the QIAquick Gel Extraction Kit (Qiagen) and sample concentration and quality verified by Nanodrop. The same amount of each purified product (50 ng for PM/EP samples; 30 ng for LP samples) was pooled according to timepoint for 454 sequencing (McGill University and Génome Québec Innovation Centre, Montréal, QC, Canada).

All of the obtained pyrosequencing data were analyzed with the Quantitative Insights Into Microbial Ecology (QIIME) workflow (Caporaso et al., 2010) following the default settings. Samples with fewer than 1500 reads of raw data were removed. Operational taxonomic units (OTUs) were then assigned according to comparison with the Greengenes database (DeSantis et al., 2006) and alpha and beta diversities were calculated. Community composition was compared using analysis of similarity (ANOSIM) within QIIME, and abundance of each OTU and beta diversity of bacterial communities was compared between diets and pregnancy stages by ANOVA using SAS (version 9.2, SAS Institute, NC, United States). *Post hoc* pairwise comparisons were performed using Tukey’s HSD method. Significance was assumed at *P* < 0.05. Differential abundance analysis was performed for the identified phylotypes using zero-inflated log-normal mixture model within Metagenomeseq package. Raw *P*-values were used due to the small sample size. Sequence data are available from the NCBI Sequence Read Archive under project ID SRP143497.

## Results

### Body Composition

There were no differences in body mass through pregnancy in the Gen0-F animals, but fat mass was raised with fructose feeding (**Table 1**). In the next generation, dams weighed more relative to Gen0 dams at the same timepoints, and fructose fed (Gen1-F) dams displayed significantly shorter small intestines relative to controls. Pancreas weight was also significantly reduced in Gen1-F dams relative to Gen1-C.

**Table 1:** Body composition measurements. All values mean ± SEM, ^∗^*p* < 0.05, ^∗∗^*p* < 0.01, one-way ANOVA with contrasts (Gen0-C vs. Gen0-F and Gen1-C vs. Gen1-F).

	Gen0-C (*n* = 9)	Gen0-F (*n* = 6)	Gen1-C (*n* = 10)	Gen1-F (*n* = 10)
Birth weight (g)	-	-	3.86 ± 0.7	3.26 ± 0.6*
Weight at GD20 (g)	422.2 ± 8.7	434.8 ± 12.3	454.4 ± 19.9	468.5 ± 19.2
Percentage fat	10.3 ± 1.4	12.9 ± 1.9	11.4 ± 0.5	16.8 ± 1.3**
Percentage lean	75.6 ± 0.9	73.3 ± 1.4	75.8 ± 0.3	71.4 ± 1.0**
Small intestinal length (cm)	124.8 ± 3.4	126.5 ± 2.6	128.5 ± 1.8	123.8 ± 0.4*
Liver (g)	18.7 ± 0.6	19.6 ± 0.8	21.1 ± 0.5	21.9 ± 1.4
Pancreas (g)	1.14 ± 0.1	1.15 ± 0.1	1.34 ± 0.1	1.08 ± 0.1*
Ileum villus height (μm)	416 ± 15	490 ± 33	662 ± 28	663 ± 38
Jejunum villus height (μm)	492 ± 42	668 ± 35*	592 ± 27	606 ± 16

### Glucose, Insulin, and Triglyceride Homeostasis Through Pregnancy

**FIGURE 2:**
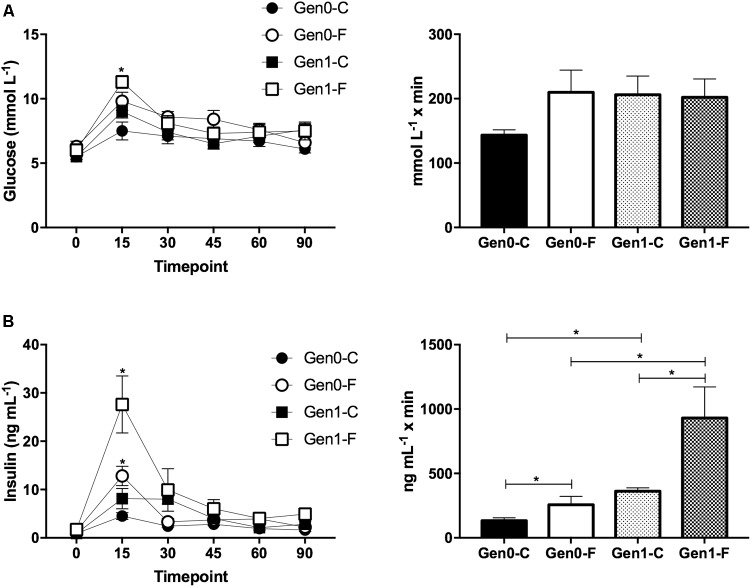
**(A)** OGTT for glucose (L) and incremental area under the curve for first and second generation dams at GD19. ^∗^*p* < 0.05 Gen1-C vs. Gen1-F, one way ANOVA with contrasts. **(B)** Plasma insulin OGTT (L) and incremental area under the curve (R) at GD19. (L) ^∗^*p* < 0.05 Gen0-C vs. Gen0-F and Gen1-C vs. Gen1-F, Unpaired *t*-test, (R) ^∗^*p* < 0.05, one way ANOVA with contrasts.

In the Gen0-F dams, plasma triglycerides were raised with fructose feeding from pre-mating (10 weeks) onward, an adaptation that was exacerbated in Gen1-F dams (**Table 2**). Plasma glucose was not different between groups throughout the OGTT in Gen0-F dams, and in Gen1-F, peak glucose was raised with fructose feeding but the area under the curve remained similar (**Figure 2A**). In contrast, plasma insulin was raised in Gen0-F dams 15 min following glucose administration and was accompanied with an increased IAUC, an adaptation that was amplified in the Gen1-F group (**Figure 2B**).

**Table 2:** Plasma glucose, insulin, and triglycerides.

	Gen0-C (*n* = 9)	Gen0-F (*n* = 6)	Gen1-C (*n* = 10)	Gen1-F (*n* = 10)
**Glucose (mmol/L)**				
Pre-diet	7.86 ± 0.6	8.01 ± 0.8	7.29 ± 0.7	7.30 ± 0.5
Pre-mating	7.99 ± 0.7	7.98 ± 1.1	7.00 ± 0.4	8.57 ± 0.9*
Early pregnancy	7.45 ± 0.9	8.27 ± 0.8*	6.80 ± 0.6	7.73 ± 0.5*
Mid pregnancy	6.53 ± 0.7	6.64 ± 0.3	5.55 ± 0.5	6.72 ± 0.8*
Late pregnancy	5.36 ± 0.6	6.32 ± 0.5	5.69 ± 0.3	6.01 ± 0.1
**Insulin (ng/mL)**				
Pre-diet	0.86 ± 0.5	0.78 ± 0.3	0.87 ± 0.5	1.03 ± 0.5
Pre-mating	0.97 ± 0.7	1.01 ± 0.5	1.37 ± 0.6	2.31 ± 1.2*
Early pregnancy	0.83 ± 0.5	1.07 ± 0.2	1.27 ± 1.0	2.76 ± 1.6*
Mid pregnancy	0.62 ± 0.2	1.47 ± 0.5	0.95 ± 0.5	2.78 ± 1.7*
Late pregnancy	0.86 ± 0.7	1.68 ± 0.4	0.86 ± 0.2	1.69 ± 0.5*
**Triglyceride (mmol/L)**				
Pre-diet	3.58 ± 1.2	3.26 ± 0.9	3.88 ± 1.5	3.71 ± 1.3
Pre-mating	4.41 ± 1.8	7.09 ± 2.2*	4.72 ± 1.0	17.58 ± 5.1*
Early pregnancy	5.19 ± 1.1	9.49 ± 3.2*	4.51 ± 1.3	14.11 ± 4.2*
Mid pregnancy	11.01 ± 4.7	18.39 ± 1.6*	10.42 ± 2.7	21.28 ± 6.7*
Late pregnancy	12.14 ± 1.0	20.32 ± 1.5*	11.42 ± 3.2	23.43 ± 3.1*

*All values mean ± SEM, ^∗^p < 0.05, (Gen0-C vs. Gen0-F and Gen1-C vs. Gen1-F), one-way ANOVA with contrasts.*

### Gut Adaptations in the Offspring and Impact of Diet and Gestation on the Diversity of Fecal Bacteria

**FIGURE 3:**
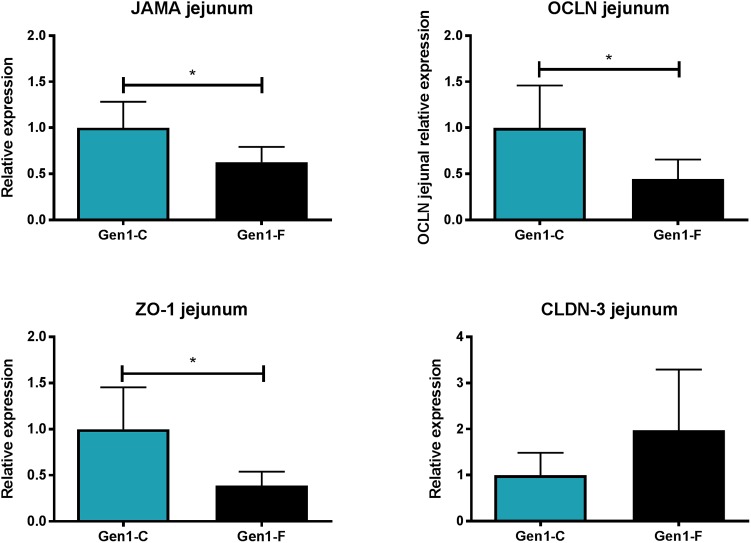
Second generation jejunum gene expression results. All values mean ± SEM, ^∗^*p* < 0.05, one way ANOVA with contrasts.

In Gen0-F dams, there was no change in expression of CLDN-3, ZO-1, JAMA, and OCLN between groups in either the jejunum or ileum (data not shown), whereas these were reduced in Gen1-F in the jejunum (**Figure 3**) but not ileum (data not shown). Villus height was significantly increased in the jejunum in Gen0-F, but unchanged between Gen1-F and Gen1-C in both ileum and jejunum (**Table 1**).

**FIGURE 4:**
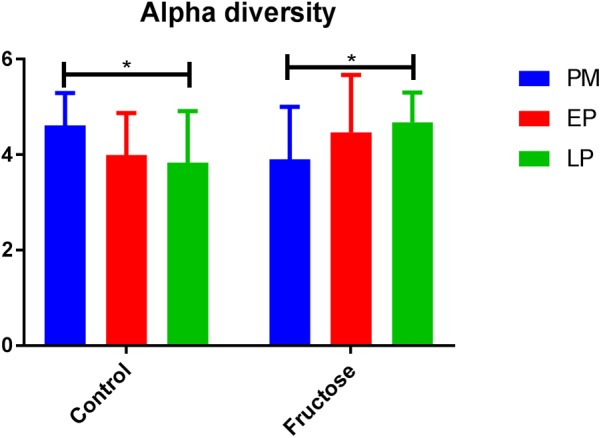
Alpha diversity (Shannon index) of bacterial communities between diets at each pregnancy timepoint.

**FIGURE 5:**
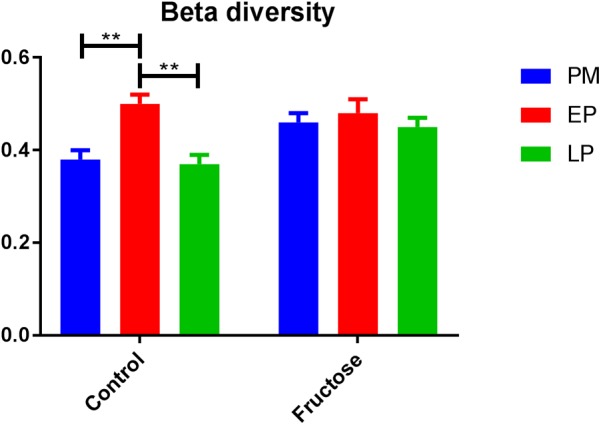
Beta diversity (Bray–Curtis dissimilarity indices) of bacterial communities between diets at each pregnancy timepoint.

With respect to total fecal bacteria, our sample sequences (1970 ± 1085 seqs/sample) covered an estimated 86–98% of the total community (Supplementary Table S2) and were represented by OTUs (1004 ± 648 OTU/sample) that were assigned to 10 phyla, 15 classes, 22 orders, 40 families, 62 genera, and 100 species. The most abundant phyla were *Bacteroidetes* and *Firmicutes* (**Table 3**). Prior to pregnancy, control animals displayed a more diverse bacterial community compared to those fed fructose (Chao1 index 85 vs. 69, respectively). Prior to, and during LP, bacterial composition was different between diet groups and alpha and beta diversity were increased in the fructose group (**Figures 4**, **5**). At all sampling points, the differentially abundant OTUs at the species level (or proportions of OTUs) between diets are presented in **Table 4**.

**Table 3:** Relative abundance of the identified bacteria phyla.

	PM		EP		LP			P		
Phyla	C	F	C	F	C	F	SEM	Period	Diet	Period ^∗^ Diet
Actinobacteria	0.20^∗^	0.43	0.45	0.64	0.39	1.00	0.12	0.363	0.157	0.733
Bacteroidetes	42.00	33.15	49.02	47.45	61.93	44.69	2.37	0.024	0.041	0.379
Cyanobacteria	0.78	0.00	0.14	0.19	0.01	0.00	0.15	0.524	0.386	0.459
Deferribacteres	0.08	0.00	0.00	0.00	0.00	0.02	0.01	0.402	0.421	0.286
Firmicutes	54.31	63.07	49.36	46.28	36.95	50.61	2.39	0.038	0.153	0.335
Proteobacteria	1.74	1.01	0.78	4.06	0.62	3.53	0.49	0.730	0.078	0.173
TM7	0.24	0.00	0.00	0.01	0.01	0.09	0.05	0.591	0.592	0.364
Tenericutes	0.40	0.15	0.08	0.11	0.08	0.00	0.03	0.006	0.087	0.200
Verrucomicrobia	0.25	2.19	0.16	1.26	0.00	0.06	0.36	0.438	0.147	0.558

*PM, pre-mate; EP, early pregnancy; LP, late pregnancy; C, Control; F, Fructose. ^∗^Data presented in proportion.*

**Table 4:** Relative abundance of the identified bacteria species.

	PM		EP		LP			P		
Species	C	F	C	F	C	F	SEM	Period	Diet	Period ^∗^ Diet
sp. of Bacteroidales	0.08	0.00	0.07	0.00	0.17	0.00	0.02	0.441	<0.001	0.269
*Bacteroides* sp.	0.92	0.01	1.82	0.01	0.66	0.04	0.25	0.477	0.027	0.595
*Parabacteroides* sp.	0.31	0.00	0.19	0.00	0.05	0.00	0.02	0.011	<0.001	0.026
*Prevotella* sp.	7.81	0.01	18.32	0.03	29.46	0.00	1.70	0.005	<0.001	0.001
sp. of family S24-7	34.12	0.47	28.44	0.22	28.83	0.23	2.16	0.225	<0.001	0.607
*Lactobacillus* sp.	5.84	0.00	6.09	0.02	5.50	0.01	0.80	0.879	<0.001	0.988
*Lactobacillus reuteri*	9.62	0.00	1.25	0.00	0.91	0.00	0.74	0.003	0.002	0.007
*Turicibacter* sp.	1.50	9.08	1.45	11.42	0.82	8.90	0.92	0.917	<0.001	0.818
sp. of Clostridiales	1.96	0.00	9.96	0.02	2.16	0.01	0.88	0.032	0.006	0.080
sp. of Lachnospiraceae	0.47	0.00	0.25	0.00	0.34	0.00	0.04	0.209	<0.001	0.280
sp. of Peptostreptococcaceae	0.01	2.17	0.11	2.86	0.03	2.03	0.19	0.642	<0.001	0.490
*Oscillospira* sp.	2.45	0.16	2.01	0.51	2.72	0.39	0.19	0.962	<0.001	0.463
*Ruminococcus* sp.	0.69	0.90	0.71	2.42	0.61	1.53	0.19	0.346	0.018	0.267
*Anaeroplasma* sp.	0.03	0.18	0.00	0.19	0.00	0.04	0.02	0.055	<0.001	0.080
sp. of order RF39	0.58	0.00	0.09	0.01	0.11	0.04	0.04	<0.001	<0.001	<0.001

*PM, pre-mate; EP, early pregnancy; LP, late pregnancy; C, control; F, fructose. ^∗^Data presented in proportion.*

**FIGURE 6:**
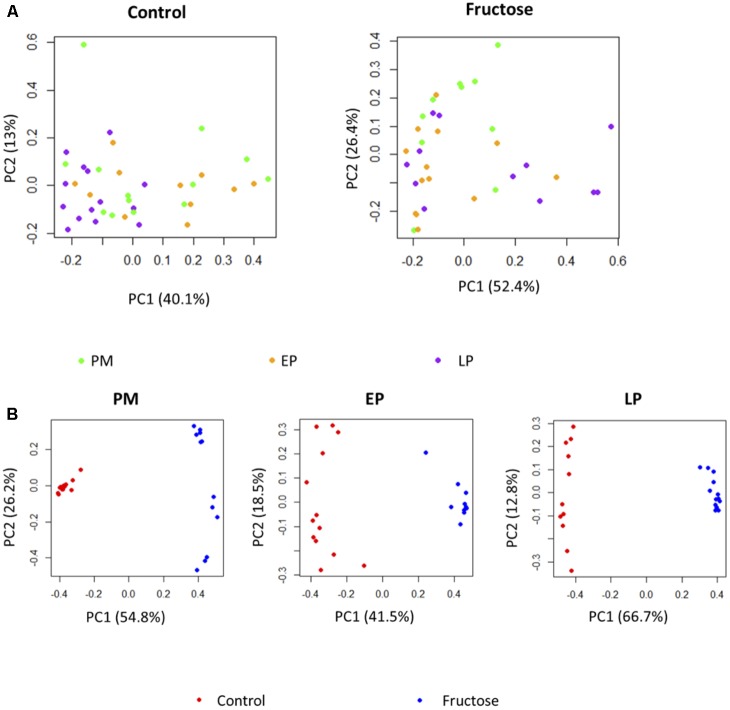
PCoA plots of bacterial profiles at species level based on Bray–Curtis dissimilarity analysis. **(A)** Pregnancy status did not affect the clustering of the bacterial profiles either under Control diet or under Fructose diet. **(B)** At each pregnancy stage, bacterial profiles clustered based on the diets they receive.

Further comparison of the effect of pregnancy stage and diet on fecal bacterial communities indicated that pregnancy stage did not have significant influence on microbial profiles (**Figure 6A**), while the different diets resulted in a strong impact (**Figure 6B**). Individual species responded to host pregnancy stage and diet in different ways. As shown in **Table 4**, only five species were differentially abundant in different pregnancy stages: along with pregnancy stage, *Parabacteroides* sp., *Lactobacillus reuteri*, and sp. of order RF39 were decreased, *Prevotella* sp. was increased, while sp. of *Clostridiales* was lowered during EP and recovered gradually during LP. The effect of diet was more obvious, with 15 species differentially abundant between control and fructose diets, among which sp. of Bacteroidales, *Parabacteroides* sp., *Lactobacillus reuteri*, and sp. of Lachnospiraceae were exclusively observed in rats given the control diet. Association analyses were performed between physiology parameters and the relative abundance of individual bacterial species; however, no correlations were seen (data not shown).

## Discussion

The present study extends our previous findings that increased consumption of fructose negatively impacts on lipid and carbohydrate metabolism due to insulin resistance (Lineker et al., 2016; Song et al., 2017). Importantly, this adverse adaptation only becomes apparent during pregnancy and is amplified in the next generation. Here we show that one factor contributing to the negative impact of fructose on maternal metabolism could be maladaptation of the microbiome. Fructose is known to affect the microbiota (Pachikian et al., 2013; Wagnerberger et al., 2013; Vos, 2014) and when the maternal microbiota is compromised with antibiotic exposure, intestinal development in the offspring is adversely affected in conjunction with the appearance of an *Enterobacteriaceae*-rich microbiome (Fåk et al., 2008). Previously, the influence of pregnancy on the microbiome has not been examined in an animal model. During pregnancy, substantial changes in maternal hormones, immune function, and metabolism occur (Mor and Cardenas, 2010; Newbern and Freemark, 2011) that could impact on the microbiome which in some human studies has been suggested to result in a more obesogenic profile (Collado et al., 2008; Moreira et al., 2012).

Birth weight was significantly reduced in Gen1-F rats; in conjunction with this, we also observed significantly shorter small intestines and dysregulation of epithelial tight junction gene expression relative to controls. This is consistent with previous work associating growth restriction and impaired small intestinal development in rats (Baserga et al., 2004), pigs (Morise et al., 2008; Wang et al., 2008), and humans (Indrio et al., 2013). The reduction we found in three of the four epithelial tight junctions studied (JAMA, OCLN, and ZO-1) in Gen1-F suggests that intestinal permeability is affected either by the fructose diet directly or through maternal gut related adaptations. There is some evidence to support both hypotheses that fructose is known to adversely affect intestinal permeability (Spruss and Bergheim, 2009) and disruption of the maternal microbiota can lead to altered offspring gut development (Fåk et al., 2008). In the case of this study, fructose is potentially adversely affecting offspring gut development via both of the above mechanisms. Further work such as a crossover design in which offspring born to fructose-fed mothers were switched to a control diet would be required to determine the relative significance of diet and the microbiota passed from the mother.

Previous work in humans has shown that regardless of pre-pregnancy body mass index, the diversity of bacterial species within an individual declines with gestation, but diversity of species between individuals increases (Koren et al., 2012). As a caveat dietary changes throughout gestation in this study were not detailed and it is known that dietary intake can adapt early in pregnancy (Rifas-Shiman et al., 2006). We observed different trends in our control animals with bacterial alpha (within individual) diversity declining by the end of pregnancy but beta (inter-individual) diversity remaining unchanged. This is potentially due to the difference in environmental exposure between rats and humans, and illustrates the potential issues of using the rat microbiome as a model for humans.

Although fructose fed animals had a less diverse (numerically but not statistically different) microbiome to begin with, the opposite trend in diversity was observed during pregnancy, with alpha diversity increasing throughout pregnancy but beta diversity remaining unchanged. Importantly, these adaptations occurred despite no change in total energy intake as the increased consumption of fructose in drinking water was compensated for by a reduced intake of food (Erlanson-Albertsson and Lindqvist, 2010; Vickers et al., 2011; Gray et al., 2013).

Chronic consumption of fructose (but not glucose) can induce endotoxemia in mice (Bergheim et al., 2008) and gene expression of claudin-4 is reduced *in vitro* with the addition of fructose (Johnson et al., 2013). In rats, disruption of the maternal microbiota has been shown to adversely affect intestinal permeability in the offspring (Fåk et al., 2008), and therefore it is possible that a microbiota deficient in *Bacteroides*, *Prevotella*, and *Ruminococcaceae* as demonstrated with fructose feeding is passed on to the offspring. There is some parallel between our study and those using *Lactobacillus* as a probiotic intervention to slow progression of type II diabetes (Yadav et al., 2007) and liver steatosis (Wagnerberger et al., 2013) in fructose fed mice and rats, suggesting that a microbiome deficient in *Lactobacillus* is part of the adverse effects caused by a high-fructose diet. However, there is currently no evidence to suggest a direct link between changes in bacterial abundance and intestinal permeability. Further studies measuring intestinal permeability directly and relevant end organs are now required to ascertain if the changes in tight junction gene expression observed in our model contribute to further adverse effects. Studies of a high fructose diet in germ-free mice, while not representing normal physiological or environmental conditions, would be worthwhile to determine whether the changes observed in this study are a direct effect of fructose or are indirectly caused by changes to the microbiome.

## Conclusion

We have shown that fructose consumption during pregnancy affects microbial diversity in the mother, with a shift toward a microbiome that is more diverse, that appears to be the opposite of changes in a normal pregnancy. Further studies into how this maternal microbiome will influence colonization in the offspring, either those continuing on a fructose supplemented diet or switching to a control diet, would be informative. Continuation of the fructose-supplemented diet by the offspring adversely affects epithelial tight junctions and therefore potentially intestinal permeability, with these effects not being apparent in the first generation.

To our knowledge, this is the first account of both these microbial changes and the offspring effects being demonstrated with a relatively low supplementation of fructose, and this may have implications for the effects of fructose consumption as part of the Western diet.

## Author Contributions

All authors contributed to the research and reviewed the manuscript. SA, AS, and RB designed the study. SA, AS, BN, and AH carried out all animal husbandry, sample collection, and laboratory work. MZ and BW carried out analysis of 16s sequencing data. SA, AS, MZ, BW, MS, and RB drafted the manuscript. All authors discussed the analysis, interpretation, and presentation of the results.

## Conflict of Interest Statement

The authors declare that the research was conducted in the absence of any commercial or financial relationships that could be construed as a potential conflict of interest.
