# Supplementary material for: High Fructose Intake During Pregnancy in Rats Influences the Maternal Microbiome and Gut Development in the Offspring
Source: Front Genet. 2018 Jun 19;9:203. doi: 10.3389/fgene.2018.00203 (PMC6018152; doi:10.3389/fgene.2018.00203)
Supplement: Supplementary file 1 [file Table_1.DOCX]

**Supplementary table 1**: PCR primer sequences

|  | **Forward** | **Reverse** | **Product size (bp)** |
| --- | --- | --- | --- |
| 60s ribosomal protein P0 (RPLP0) | GATGCCCAGGGAAGACAG | CACAATGAAGCATTTTGGGTAG | 91 |
| Glyceraldehyde-3-phosphate dehydrogenase (GAPDH) | TGGAGTCTACTGGCGTCTT | TGTCATATTTCTCGTGGTTCA | 138 |
| Zonulin-1 (ZO-1) | gcatgtagacccagcaaagg | ggttttgtctcatcatttcctca | 65 |
| Claudin-3 (CLDN-3) | GGACCCAGTTCACCTTTCC | GCCTCTAATCCCTCATTTCACA | 188 |
| Occludin (OCLN) | atctagagcctggagcaacg | gtcaaggctcccaagacaag | 63 |
| Junctional adhesion molecule A (JAMA) | gcctatagccgtggatactttg | acgaggtctgtttgaattctcc | 112 |
| 16s rRNA 27F / 519R | AGAGTTTGATCMTGGCTCAG | GWATTACCGCGGCKGCTG | 530 |
