# Supplementary material for: High Fructose Intake During Pregnancy in Rats Influences the Maternal Microbiome and Gut Development in the Offspring
Source: Front Genet. 2018 Jun 19;9:203. doi: 10.3389/fgene.2018.00203 (PMC6018152; doi:10.3389/fgene.2018.00203)
Supplement: Supplementary file 2 [file Table_2.DOCX]

**Supplementary Table 2**. Summary of bacterial community within each sample.

| Sample ID | Chao1^a^ | Good’s coverage^b^ | Observed species^c^ | Shannon^d^ | Simpson^e^ |
| --- | --- | --- | --- | --- | --- |
| 118pm | 221 | 0.931 | 142 | 5.46 | 0.956 |
| 120pm | 253 | 0.930 | 145 | 5.44 | 0.945 |
| 121pm | 143 | 0.961 | 85 | 3.82 | 0.785 |
| 122pm | 173 | 0.932 | 120 | 4.98 | 0.920 |
| 123pm | 147 | 0.960 | 97 | 4.41 | 0.856 |
| 124pm | 138 | 0.937 | 74 | 4.45 | 0.912 |
| 125pm | 131 | 0.964 | 84 | 3.91 | 0.828 |
| 126pm | 100 | 0.923 | 62 | 4.21 | 0.883 |
| 127pm | 153 | 0.958 | 107 | 4.84 | 0.919 |
| 128pm | 121 | 0.936 | 78 | 4.47 | 0.906 |
| 129pm | 125 | 0.921 | 100 | 5.43 | 0.953 |
| 130pm | 145 | 0.917 | 97 | 5.32 | 0.940 |
| 131pm | 90 | 0.973 | 51 | 2.65 | 0.671 |
| 133pm | 188 | 0.919 | 124 | 5.36 | 0.931 |
| 135pm | 63 | 0.982 | 41 | 2.28 | 0.627 |
| 137pm | 154 | 0.956 | 100 | 4.31 | 0.870 |
| 138pm | 119 | 0.967 | 69 | 3.71 | 0.834 |
| 139pm | 136 | 0.903 | 84 | 5.27 | 0.948 |
| 141pm | 127 | 0.960 | 85 | 3.86 | 0.822 |
| 143pm | 108 | 0.969 | 64 | 3.59 | 0.819 |
| 144pm | 142 | 0.926 | 99 | 5.21 | 0.948 |
| 145pm | 65 | 0.913 | 38 | 4.03 | 0.889 |
| 146pm | 60 | 0.983 | 36 | 2.15 | 0.633 |
| 147pm | 87 | 0.954 | 66 | 3.87 | 0.845 |
| 149pm | 87 | 0.875 | 50 | 4.73 | 0.941 |
| 151pm | 116 | 0.967 | 67 | 3.59 | 0.824 |
| 118EP | 96 | 0.975 | 63 | 3.72 | 0.847 |
| 120EP | 146 | 0.961 | 91 | 4.55 | 0.916 |
| 121EP | 87 | 0.955 | 44 | 2.89 | 0.738 |
| 122EP | 303 | 0.910 | 168 | 5.14 | 0.904 |
| 123EP | 177 | 0.934 | 103 | 5.23 | 0.947 |
| 124EP | 149 | 0.93 | 98 | 4.81 | 0.892 |
| 125EP | 128 | 0.963 | 93 | 4.09 | 0.853 |
| 126EP | 147 | 0.859 | 78 | 5.02 | 0.928 |
| 127EP | 225 | 0.935 | 119 | 3.92 | 0.828 |
| 128EP | 186 | 0.950 | 108 | 4.74 | 0.916 |
| 131EP | 90 | 0.874 | 54 | 4.83 | 0.943 |
| 132EP | 149 | 0.960 | 92 | 4.28 | 0.878 |
| 134EP | 63 | 0.982 | 37 | 1.26 | 0.303 |
| 137EP | 188 | 0.952 | 89 | 3.56 | 0.762 |
| 138EP | 97 | 0.939 | 69 | 4.39 | 0.907 |
| 139EP | 191 | 0.944 | 110 | 3.75 | 0.788 |
| 141EP | 139 | 0.862 | 85 | 5.39 | 0.955 |
| 144EP | 129 | 0.896 | 85 | 5.01 | 0.936 |
| 145EP | 118 | 0.943 | 80 | 4.88 | 0.940 |
| 146EP | 258 | 0.907 | 116 | 5.05 | 0.932 |
| 147EP | 132 | 0.925 | 79 | 4.63 | 0.903 |
| 148EP | 85 | 0.976 | 55 | 2.14 | 0.505 |
| 149EP | 87 | 0.977 | 58 | 3.30 | 0.765 |
| 120LP | 139 | 0.941 | 65 | 3.59 | 0.774 |
| 121LP | 88 | 0.956 | 57 | 4.45 | 0.922 |
| 122LP | 73 | 0.919 | 54 | 5.01 | 0.956 |
| 123LP | 212 | 0.901 | 120 | 5.26 | 0.936 |
| 124LP | 121 | 0.947 | 73 | 4.42 | 0.901 |
| 126LP | 161 | 0.880 | 87 | 4.97 | 0.901 |
| 127LP | 115 | 0.948 | 83 | 4.01 | 0.783 |
| 129LP | 141 | 0.933 | 90 | 4.77 | 0.922 |
| 130LP | 102 | 0.928 | 51 | 3.86 | 0.852 |
| 132LP | 102 | 0.959 | 70 | 4.57 | 0.907 |
| 133LP | 66 | 0.953 | 49 | 4.25 | 0.904 |
| 134LP | 99 | 0.961 | 50 | 3.48 | 0.829 |
| 135LP | 118 | 0.935 | 73 | 4.64 | 0.921 |
| 137LP | 56 | 0.973 | 43 | 1.85 | 0.400 |
| 138LP | 127 | 0.943 | 92 | 5.05 | 0.936 |
| 139LP | 85 | 0.960 | 57 | 3.66 | 0.827 |
| 141LP | 142 | 0.878 | 102 | 5.72 | 0.963 |
| 142LP | 106 | 0.88 | 60 | 4.53 | 0.903 |
| 143LP | 130 | 0.898 | 84 | 5.33 | 0.951 |
| 144LP | 93 | 0.958 | 43 | 2.01 | 0.443 |
| 145LP | 71 | 0.942 | 53 | 3.52 | 0.786 |
| 146LP | 129 | 0.866 | 57 | 4.58 | 0.929 |
| 147LP | 153 | 0.935 | 92 | 5.23 | 0.956 |
| 149LP | 105 | 0.953 | 67 | 4.24 | 0.904 |

^a^ Chao1: estimated species richness.

^b^ Good’s coverage: Good’s coverage for an OTU definition.

^c^ Observed species: unique OTU numbers.

^d^ Shannon: Shannon diversity index.

^e^ Simpson: Simpson’s diversity index.
